# Assessment of Overweight, Obesity, Central Obesity, and Type 2 Diabetes among Adolescents in Qatar: A Cross-Sectional Study

**DOI:** 10.3390/ijerph192114601

**Published:** 2022-11-07

**Authors:** Sohaila Cheema, Amit Abraham, Katie G. El-Nahas, Rasha Abou-Amona, Abdulla O. Al-Hamaq, Patrick Maisonneuve, Karima Chaabna, Albert B. Lowenfels, Ravinder Mamtani

**Affiliations:** 1 Institute for Population Health, Weill Cornell Medicine-Qatar, Education City, Qatar Foundation, Doha P.O. Box 24144, Qatar; 2 Qatar Diabetes Association, Doha P.O. Box 752, Qatar; 3 Division of Epidemiology and Biostatistics, IEO European Institute of Oncology IRCCS, 20141 Milan, Italy; 4 Department of Surgery and Department of Family Medicine, New York Medical College, Valhalla, New York, NY 10595, USA

**Keywords:** obesity, type 2 diabetes mellitus, Middle East, epidemiology

## Abstract

Qatar has a high obesity and type 2 diabetes mellitus (T2DM) burden. This study aimed to (1) determine the prevalence of overweight, obesity, and T2DM in 13–17-year-old adolescents and (2) evaluate associations with adolescents’ lifestyle and breastfeeding history, parental weight, and familial T2DM history. A cross-sectional study (double-stage cluster sampling) was conducted in 2018–2020 using a self-administered parental and adolescent questionnaire. In the results, 23.4% of the adolescents (107/459) were overweight; 19.9% (91/459) were obese; and 37.6% (171/459) had evidence of central obesity. Random blood sugar (RBS) was suggestive of prediabetes (≥140 mg/dL) for 23 (5.0%) adolescents and T2DM (≥200 mg/dL) for none. In multivariable analysis, obesity was significantly associated with no breastfeeding (OR = 3.17, 95% CI: 1.09–9.26) compared to breastfed adolescents for ≥6 months, with first-degree family history of T2DM (OR = 2.27; 95% CI: 1.22–4.27), with maternal obesity (OR = 2.40; 95% CI: 1.01–5.70), and with acanthosis nigricans in adolescents (OR = 19.8; 95% CI: 8.38–46.9). Central obesity was significantly associated with maternal obesity (OR = 2.21; 95% CI: 1.14–4.27) and with acanthosis nigricans (OR = 3.67; 95% CI: 1.88–7.18). Acanthosis nigricans (OR = 4.06; 95% CI: 1.41–11.7) was the only factor associated with elevated RBS. Addressing future disease burden among adults in Qatar will require extensive health and well-being programs, focused on healthy lifestyles and behaviors such as nutritious diets, physical activity, stress management, and self-care.

## 1. Introduction

The World Health Organization (WHO) has recognized that many chronic conditions such as overweight, obesity, type 2 diabetes mellitus (T2DM), and cardiovascular disease (CVD) are predominantly preventable [1]. Their prevalence is widespread and increasing. The proportion of overweight children between 5 and 19 years of age, for example, nearly doubled from 10.3% in 2000 to 18.4% in 2016 [2]. Overweight and obese children and adolescents are at risk of developing both short and long-term health consequences of their weight excess, such as sleep apnea, T2DM, certain types of cancer, hypertension, CVD, asthma, and liver disease across the lifespan [3]. Overweight and obesity in children and adolescents are likely to continue into adulthood [1,4]. The link between overweight and obesity and the development of non-communicable chronic diseases, especially T2DM and CVD at a younger age has been clearly established [1,5]. Not only do children and adolescents suffer the physical ramifications of living overweight or obese, but they must also deal with the stigma and mental health consequences, including, but not limited to, poor self-esteem and depression [2]. Being overweight and obese has also been linked to poor academic performance [3]. Central obesity, as measured by waist circumference, has been associated with enhanced CVD and other sequelae risks in adolescents, and some have argued that central obesity is a better predictor of long-term complications than obesity [6,7]. Therefore, overweight and obesity in the younger population constitute a serious public health problem.

Oxidative stress and chronic inflammation contribute to the development of insulin resistance, leading to the development of prediabetes, T2DM, obesity, and CVD [8,9]. For instance, obese individuals have a higher risk of developing CVD than normal-weight individuals, and among the obese population, the risk is higher in metabolically unhealthy individuals compared to metabolically healthy individuals. Oxidative stress plays a key role in the pathogenesis of CVD [10]. The release of reactive oxidative species can result in mitochondrial and endothelial dysfunction, which is the underlying mechanism for CVD pathophysiology [11].

T2DM has been recognized as a rapidly spiraling concern among children and adolescents. Many of the long-term complications of T2DM overlap with those of overweight and obesity. T2DM that presents in adolescents typically responds less promisingly to diabetic medication and progresses more swiftly than adult-onset T2DM [12]. Further, T2DM is more likely to result in CVD, microvascular complications, and overall mortality compared to type 1 diabetes in the same cohort [12,13,14]. It is important to diagnose adolescents with T2DM as early as possible. Acanthosis nigricans, defined as hyperpigmentation of the skin in areas such as the back of the neck, axilla, and groin, can sometimes be a presenting sign of T2DM. It is commonly seen in cases of T2DM and obesity, especially among the youth [15]. Prediabetes, sometimes referred to as impaired glucose tolerance or impaired fasting glucose, is predictive of the development of T2DM and its subsequent complications [12,13]. The prevalence of prediabetes among adolescents is elevated in high-income countries such as the USA, where it is estimated to be 18% [16], compared to Qatar, another high-income country where an earlier study determined it was 4.2% in 2012 [17]. 

In countries undergoing rapid socio-economic development, the prevalence of overweight and obese children and adolescents, as well as prediabetes and T2DM has continued to increase [1]. Previously a burden of the wealthy, T2DM, overweight, and obesity are now common across socioeconomic strata due to the easy availability, accessibility, and low economic costs of poor quality, highly processed foods and beverages, and reduced reliance on wholesome food and traditional, healthy cooking practices [2]. This is also true for the high-income Middle East countries with oil and gas-based economies such as Qatar. A study from Qatar published in 2014 reported that 46% and 44% of male and female adolescent students were overweight or obese [17]. Furthermore, the study also reported that 4.2% of these students aged 11–18 years were pre-diabetic [17]. This is highly concerning pertaining to a country with a high prevalence of adult T2DM at 17% [18]. 

Many factors (e.g., lifestyle habits and family history) have been identified to increase the risk of overweight/obesity and T2DM in adolescents. This includes pre- and peri-natal factors such as maternal health and obesity, early childhood factors such as birthweight and breastfeeding practices, cigarette smoke and other environmental exposures, adolescent physical activity and nutrition, cultural practices and the like [19,20,21,22,23,24,25,26]. Risk factors for this growing epidemic of overweight/obesity and T2DM need to be assessed regularly; as such, up-to-date public health interventions can be implemented. 

The primary objective of this study is to assess the prevalence of adolescents’ overweight, obesity, central obesity, prediabetes, and T2DM, and the secondary study objective is to evaluate the association of adolescents’ overweight, obesity, central obesity, prediabetes, and T2DM with adolescents’ eating and physical activity habits, parents’ weight status and diabetes history, and breastfeeding history.

## 2. Materials and Methods

### 2.1. Study Design

A cross-sectional study was conducted between 15 March 2018, and 15 January 2020. This study is reported using the Strengthening the Reporting of Observational Studies in Epidemiology (STROBE) guidelines for reporting cross-sectional studies (Supplementary checklist) [27].

### 2.2. Participants, Eligibility Criteria, and Study Setting

Adolescents aged between 13 and 17 years old attending governmental and private schools in Qatar were eligible for enrolment in the study. Adolescents already diagnosed with type 1 diabetes were excluded, as were those adolescents who were not fluent in either Arabic or English.

### 2.3. Primary and Secondary Outcomes

The study’s primary outcomes were the prevalence of overweight, obesity, central obesity, T2DM, and prediabetes among adolescents in Qatar. 

Body mass index (BMI) was defined as body weight (kg) divided by height squared (m^2^). Age and sex specific cut-offs as recommended by the WHO were used to define overweight and obese adolescents. Overweight was defined as BMI-for-age > 1 standard deviation (SD; equivalent to BMI > 25 kg/m^2^ at 19 years) and obesity as BMI-for-age >2 SD (equivalent to BMI > 30 kg/m^2^ at 19 years) [28,29]. Central obesity was defined according to international waist circumference (WC) percentile cut-offs for central obesity in children and adolescents aged 6 to 18 years proposed by Xi et al. [30]. Random blood sugar (RBS) was measured during a single encounter with a pin prick. We used 140 mg/dL as the cut-off to identify adolescents who may have prediabetes, as has been done in previous studies [31,32], and ≥200 mg/dL as the cut-off to identify adolescents who may have T2DM, in line with recommendations by the American Diabetes Association (ADA) and the Centers for Disease Control and Prevention (CDC) [33,34].

### 2.4. Sample Size

Data analysis was done on 459 adolescents based in 24 governmental and private schools in Qatar. Assuming that the prevalence of overweight or obesity among adolescents in Qatar is around 45%, a sample of 377 participants produces a two-sided 95% confidence interval with a width equal to 0.10, using the Wilson score interval method. Accounting for a 10% of attrition and incomplete data, the total sample size was estimated to 419. Once we reached the target sample population, we decided to continue recruitment to enhance the sample population size.

We used double-stage cluster sampling. Schools in Qatar were randomly selected from all Qatar schools with intermediate levels. The Ministry of Education sent a memo to the selected schools to participate in the study. Trained Qatar Diabetes Association staff approached the selected schools. The selected school administration was asked to share a list of all adolescents within the 13–17-year age group; adolescents were randomly selected from the list through systematic random sampling. Once the adolescents were selected, they were informed regarding the study details by the school administration, then parental consent and adolescent assent were requested. 

### 2.5. Ethical Approval and Informed Consent and Assent

The study was conducted in accordance with ethical standards required by both Qatar and the USA for the protection of human participants. The Institutional Review Board (IRB) of Weill Cornell Medicine—Qatar reviewed the proposal, and the study received expedited approval (IRB number 14-00004).

A parental letter containing the parental consent document and the parental questionnaire was sent home with each adolescent. The parental consent document explained the study aim, methodology, benefits, and risks, and clearly mentioned that parents themselves must agree to participate and agree to their child’s participation in the study. The parental consent was written in English and Arabic, and then professionally translated into two other languages—Hindi and Urdu. More than two authors were fluent in each of the languages, which facilitated the parents’ understanding of the ethical considerations of the study. The professional translators (Babylon Translation Company, Doha, Qatar) were not involved in any step beyond translating into Hindi and Urdu. Documentation of parental consent was ensured before administering the questionnaire and measurements to the student. Additionally, each adolescent signed an assent form (in English or Arabic) to confirm his/her willingness to participate in the study. 

### 2.6. Variables and Measurements

The parental questionnaire contained questions about the diabetes (type 1 and type 2) status of family members, as well as relevant information relating to childbirth weight, pregnancy, breastfeeding, exposure to secondhand smoke, both parents’ height and weight, and familial history of diabetes. 

The adolescent questionnaire contained questions related to adolescent demographics, eating habits, physical activity, sedentary behavior, and screen use. Trained staff measured and recorded height, weight, and waist circumference performed a skin examination, and collected random blood sugar levels on adolescents during school hours. A skin examination was performed behind the neck to check for acanthosis nigricans if the student consented to the procedure. If the student did not consent to the skin examination, the staff showed him/her pictures of acanthosis nigricans and asked them if they had noticed similar patterns on their skin. 

### 2.7. Statistical Analyses

We analyzed the association between adolescent demographics, family history, dietary pattern, food consumption, physical activity, and sedentary behavior and BMI (normal weight, overweight, obese), WC (normal, central obesity), and RBS (<140, ≥140 mg/dL). We used the Chi-square test or Fisher’s exact test for categorical variables and the Mantel–Haenszel test trend for ordered variables. Multivariable logistic regression for dichotomous outcomes (WC, RBS) and ordinal logistic regression for ordinal dependent variables (BMI) was used to identify significant predictors of obesity, central obesity, and latent prediabetes after adjustment for gender, country of citizenship, and other factors associated with these outcomes at univariable analysis. Data analysis was conducted using the SAS software version 9.4 (Cary, NC, USA). All tests were two-sided and p-values less than 0.05 were considered statistically significant.

## 3. Results

The final analysis is based on data from 459 adolescents (of which 53.4% are girls) aged from 13 to 17 years (mean age 14.8 years) from 25 different schools. Only a quarter of the adolescents (117; 25.5%) are Qatari citizens; 152 (33.1%) adolescents are citizens from other Arab countries, 118 (25.7%) from South Asia, 53 (11.5%) from East Asia or the Pacific region, and 16 (3.5%) from countries in other regions (Table 1).

Almost a quarter of the adolescents (*n* = 107; 23.4%) were overweight and 19.9% (*n* = 91) were obese, according to the WHO BMI-for-age cut-offs. The respective proportions were similar for boys (25.2% and 22.4%) and girls (22.1% and 18.0%; *p* = 0.27), but varied significantly according to the country of citizenship, with a higher proportion of overweight or obese adolescents from Qatar and a lower proportion of adolescents originating from other Arab countries or South Asia. A total of 171 (37.6%) adolescents had evidence of central obesity, assessed using international waist circumference percentile cut-offs. The proportion was significantly higher in girls (49.6%) than boys (24.4%; *p* < 0.0001) and varied according to country of citizenship, with a higher proportion in South Asian adolescents. Random blood sugar was ≥140 mg/dL for 23 (5.0%) adolescents, with no difference according to sex or country of citizenship (Table 1). No adolescent had a glucose value ≥ 200 mg/dL which would indicate T2DM.

The association between adolescents’ characteristics and BMI (normal, overweight, obese), waist circumference (normal, central obesity), and random blood sugar (<140, ≥140 mg/dL) is presented in Table 2. Overweight or obesity was significantly associated with the absence of breastfeeding, with a higher proportion for adolescents who were not breastfed (12.0% overweight and 44.0% obese) than for adolescents who were breastfed for less than 6 months (19.8% overweight and 30.2% obese) and adolescents who were breastfed for 6 months or more (24.7% overweight and 15.1% obese) (*p* = 0.0007). Being overweight or obese was also significantly associated with a family history of T2DM in first degree relatives (*p* = 0.001), or in any of the mother’s relatives (*p* = 0.01) but not in any of the father’s relatives (*p* = 0.84). A strong association was also observed between adolescents’ BMI and the BMI of the mother (*p* = 0.004), but the association was only of borderline significance with the BMI of the father (*p* = 0.08). Finally, the presence of acanthosis nigricans was observed in 56 (12.2%) of the adolescents and was strongly correlated with obesity (*p* < 0.0001) (Table 2). Elevated waist circumference indicative of central obesity was associated with the mother’s BMI (*p* = 0.007) and the presence of adolescent acanthosis nigricans (*p* = 0.0005), which was the only factor significantly associated with elevated random blood sugar (*p* = 0.04) at univariate analysis (Table 2).

We also analyzed the association between BMI, WC, and RBS and adolescents’ dietary patterns (Supplementary Table S1), food consumption (Supplementary Table S2), and physical activity or sedentary behavior (Supplementary Table S3), but few associations were observed. Adolescents with elevated random blood sugar reported significantly eating snacks more frequently between meals (*p* = 0.04) and at late hours of the night (*p* = 0.04). An unexpected inverse association was found between the consumption of snacks such as chips, chocolates, biscuits, or candies and BMI (*p* = 0.004). Increased BMI was significantly associated with increased time spent watching TV or using a computer, a tablet, or a mobile phone (*p* = 0.03). Finally, central obesity was inversely associated with engagement in any sport or fitness activity (*p* = 0.0003).

In multivariable analysis adjusted for gender and country of citizenship, obesity was significantly associated with the absence of breastfeeding (OR = 3.17, 95% 1.09–9.26; *p* = 0.03) compared to adolescents who were breastfed for 6 or more months, with first degree family history of T2DM (OR = 2.27; 95% CI 1.22–4.27; *p* = 0.01), with obesity of the mother (OR = 2.40; 95% CI 1.01–5.70; *p* = 0.048) and with acanthosis nigricans (OR = 19.8; 95% CI 8.38–46.9; *p* > 0.0001). Central obesity is significantly associated with maternal obesity (OR = 2.21; 95% CI 1.14–4.27; *p* = 0.02) and with acanthosis nigricans (OR = 3.67; 95% CI 1.88–7.18; *p* = 0.0001). Acanthosis nigricans (OR = 4.06; 95% CI 1.41–11.7; *p* = 0.009) remains the only factor associated with elevated random blood sugar (Table 3).

## 4. Discussion

This study estimates the prevalence of overweight and obesity at 23.4% and 19.9% respectively among 459 adolescents (13–17 years old) in Qatar (2018–2020). While no statistically significant difference was identified between boys and girls for overweight and obesity, central obesity prevalence was double among girls. Additionally, Qatari adolescents had a higher proportion of overweight and obesity when compared to other nationalities, while South Asian adolescents had a higher proportion of central obesity. The study identified no adolescent with an RBS ≥ 200 mg/dL and estimated the prevalence of prediabetes (using RBS > 140 mg/dL in alternative to fasting blood sugar) at 5% with no difference between boys and girls. Previous studies have reported conflicting results–some have observed that boys have higher prevalence rates of T2DM, while others report a higher prevalence among girls [12,35].

Our findings are similar to those reported in previous studies conducted among adolescents in Qatar in 2012 [17] and 2015–2016 [36], suggesting that there was no change in overweight and obesity prevalence among adolescents in the last decade. During the period 2015–2016, the prevalence of overweight was estimated at 24.2% and 21.3% among 10–14 years old and 15–19 years old adolescents in Qatar, and the prevalence of obesity was 25.2% and 20.8%, respectively [36]. Additionally, our findings indicate that Qatari adolescents have a higher prevalence of overweight and obesity when compared to other nationalities, a finding reported in a previous study [36]. Overweight and obesity among adolescents are a concerning public health problem as they increase the risk in this population of suffering from premature CVD [37]. Improving the walkability options in neighborhoods and increasing road traffic safety can be effective to address overweight and obesity among adolescents [38]. A built environment characterized by low traffic safety regardless of other walkability-related features has been reported as obesogenic in this population [38]. Easy accessibility to unhealthy food, recently enhanced with online food delivery services, is associated with increased food consumption [39]. Policy measures restricting access and commercialization of unhealthy food and snacks are also deemed necessary.

A substantial proportion of adolescents in Qatar had central obesity and this appears to be higher as compared to neighboring countries. For instance, central obesity prevalence among adolescents (12–18 years old) was 26.5% in Saudi Arabia (2019) [40]. Adolescents with central obesity are at higher risk of cardiometabolic risk factors, such as elevated triglycerides and blood pressure and reduced high-density lipoprotein [41]. Central obesity is a marker of insulin resistance, the underlying pathogenic disorder for the development of the metabolic syndrome and T2DM [42]. Consequently, central obesity is a strong risk factor for metabolic syndrome [43] and is one of the criteria for its diagnosis [44]. In Saudi Arabia, where central obesity prevalence is lower than in Qatar [40], metabolic syndrome among adolescents (12–18 years old) was four times higher than the global estimate (20.6% in 2019 [40] vs. 5% in 2020 [44]), suggesting that metabolic syndrome is likely also of concern among adolescents in Qatar. Additionally, an increased level of blood glucose, which is a sign of prediabetes, is also a sign of metabolic syndrome [45]. We estimated prediabetes prevalence at 5%, which is notably similar to the prevalence estimated in 2012 in adolescents (11–18 years) at 4.2% [17]. Metabolic syndrome [46] and prediabetes [47] both increase the risk of CVD and T2DM. Adolescent T2DM often results in worse outcomes than type 1 diabetes, with both higher rates and earlier onset of macro- and microvascular disease [12]. Our findings point out that central obesity, metabolic syndrome, and prediabetes are likely prevalent and of concern among adolescents in Qatar. This study highlights the potential disease burden among future adults in Qatar, if central obesity, metabolic syndrome, and prediabetes are not adequately addressed among adolescents at the current time. This emphasizes the importance of early diagnosis of T2DM, and parents and educators ought to be aware that acanthosis nigricans is prevalent in adolescents in Qatar and is a potential presenting sign of T2DM that needs to be clinically evaluated, when detected.

Central obesity among adolescents in Qatar was associated with an unhealthy diet (i.e., intake of fast food, sweets and candy, French fries, and cakes and donuts) [48]. In our study, adolescents with elevated random blood sugar reported snacking more frequently between meals and late-night hours. This increased snacking could be driven by hyperinsulinemia, which is usually associated with glucose intolerance. Late intake of food is known to disrupt the body’s circadian rhythms, affecting insulin sensitivity and predisposing to glucose intolerance [49]. The inverse association between snacking and BMI could be explained by under- or misreporting often encountered by obese adolescents [50,51].

The prevalence of physically active youth in Qatar (≤19 years) was estimated at 15% in 2011 [52]. This prevalence was lower than that in the Middle East and North Africa, estimated at 25.6%, and in the Gulf Cooperation Council, estimated at 33.3% [52]. Implementing evidence-based public health interventions aiming to change adolescents’ lifestyles by improving their knowledge and attitude towards healthy eating and physical activity is required. Additionally, higher central obesity prevalence among adolescent girls, also previously identified in Qatar in 2012 [17] and 2013–2014 [48] highlights the urgent need for public health interventions specifically targeting adolescent girls. 

The only identified significant protective factor associated with BMI was being breastfed for more than six months. Our findings emphasize that adolescents who were breastfed for a minimum of six months were less likely to be overweight or obese. A longitudinal study suggests that breastfeeding for at least six months confers a decreased risk of childhood obesity by 42% (OR: 0.58, 95% CI: 0.36–0.94) [53]. Breastfeeding for a minimum of six months was reported low in the Eastern Mediterranean region (20.5–33.0%) [54,55]. Therefore, encouraging breastfeeding for more than six months can help to address the burden of overweight and obesity among adolescents. Identified risk factors associated with obesity among adolescents were a family history of T2DM in first degree relatives and obesity of the mother. Maternal obesity was also associated with adolescent central obesity. Public health interventions aiming to address overweight and obesity among adolescents should target families as parents influence and control their children’s energy-balance behaviors, including diet, physical activity, media use, and sleep [56].

The strength of this study is that data were collected from a population sample selected using a double-stage cluster sampling, which is the appropriate probability sampling method to study the large population of adolescents residing in Qatar [57]. Therefore, this sampling method ensured the high external validity of our study, which allows the generalizability of our findings to the adolescent population of Qatar. Our study is not without limitations. One limitation of this study was the use of RBS as a casual blood glucose test to facilitate the accomplishment of the study instead of fasting blood sugar, which measures blood glucose after not eating for at least eight hours. Fasting blood sugar is usually the first test done to check prediabetes and T2DM [58]. However, RBS is a useful measure as glucose levels in healthy individuals should not vary widely throughout the day [58]. Another limitation may pertain to the accuracy of the self-reported information.

## 5. Conclusions

Overall, our study estimated that the proportion of adolescents who are overweight or obese is approaching 50%, and prediabetes prevalence is 5%. Preventing the future burden of disease among adults in Qatar will require an extensive well-being education program, focused on healthy lifestyles such as healthy diet and physical activity [17]. The study results will contribute to developing recommendations and guide policymakers to take necessary actions to prevent and/or delay the onset of T2DM and other co-morbid conditions such as hypertension and CVD. Multi-disciplinary treatment and preventive approaches incorporating behavioral, psychological, and environmental considerations are needed to arrest this population’s rapid rise in overweight, obesity, and T2DM. Overweight and obesity among adolescents should be addressed upstream by encouraging breastfeeding for more than six months and by developing evidence-based, comprehensive, tailored family-based interventions supporting breastfeeding practices. Policies conducive to a healthy food environment are also needed to help achieve sustainable behavioral changes. Additionally, policies improving the walkability in Qatar’s neighborhoods and increasing road traffic safety can be an effective intervention to address overweight and obesity among adolescents [38]. This study can help inform future decision-making for the development of family-based interventions that incorporate the family as a unit rather than an individual adolescent to prevent obesity among children and adolescents. 

## Figures and Tables

**Table 1:** Proportion of adolescents with increased body mass index, waist circumference, or elevated random blood sugar.

	All	BMI for Age Z-Score (WHO)				Waist Circumference (WC)			Random Blood Sugar (RBS)		
	Subjects	Normal	Overweight	Obese		Normal	Central Obesity		<140 mg/dL	≥140 mg/dL	
	N (% Col)	N (% Row)	N (% Row)	N (% Row)	*p*-Value	N (% Row)	N (% Row)	*p*-Value	N (% Row)	N (% Row)	*p*-Value
All adolescents	459 (100.0)	260 (56.8)	107 (23.4)	91 (19.9)		284 (62.4)	171 (37.6)		436 (95.0)	23 (5.0)	
Age											
13 years	91 (19.8)	51 (19.6)	23 (21.5)	17 (18.7)	0.57	57 (20.1)	33 (19.3)	0.30	87 (20.0)	4 (17.4)	0.67
14 years	109 (23.8)	66 (25.4)	28 (26.2)	15 (16.5)		74 (26.1)	35 (20.5)		106 (24.3)	3 (13.0)	
15 years	112 (24.4)	67 (25.8)	21 (19.6)	24 (26.4)		71 (25.0)	39 (22.8)		104 (23.9)	8 (34.8)	
16 years	89 (19.4)	44 (16.9)	21 (19.6)	23 (25.3)		52 (18.3)	36 (21.1)		84 (19.3)	5 (21.7)	
17 years	58 (12.6)	32 (12.3)	14 (13.1)	12 (13.2)		30 (10.6)	28 (16.4)		55 (12.6)	3 (13.0)	
Gender											
Boys	210 (45.8)	110 (52.4)	53 (25.2)	47 (22.4)	0.27	158 (75.6)	51 (24.4)	**<0.0001**	201 (95.7)	9 (4.3)	0.53
Girls	245 (53.4)	146 (59.8)	54 (22.1)	44 (18.0)		122 (50.4)	120 (49.6)		231 (94.3)	14 (5.7)	
Country of citizenship											
Qatar	117 (25.5)	59 (50.4)	23 (19.7)	35 (29.9)	**0.0004**	84 (71.8)	33 (28.2)	**0.001**	111 (94.9)	6 (5.1)	0.87
Other Arab country	152 (33.1)	90 (59.2)	39 (25.7)	23 (15.1)		101 (66.9)	50 (33.1)		144 (94.7)	8 (5.3)	
South Asia	118 (25.7)	72 (61.5)	34 (29.1)	11 (9.4)		54 (46.6)	62 (53.5)		113 (95.8)	5 (4.2)	
East Asia and Pacific	53 (11.5)	28 (52.8)	6 (11.3)	19 (35.9)		32 (61.5)	20 (38.5)		49 (92.5)	4 (7.5)	
Other countries	16 (3.5)	9 (56.2)	4 (25.0)	3 (18.8)		11 (68.7)	5 (31.3)		16 (100.0)	0 (0.0)	

Percentages may not add to 100% as data are missing for some variables (number of missing values: “Gender” *n* = 4; “Country of citizenship” *n* = 3; “BMI” *n* = 1; “Waist circumference” *n* = 4). *p*-values are obtained using the chi-square test or the Fisher’s exact test. Significant *p*-values are reported in bold format.

**Table 2:** Association between adolescents’ characteristics and increased body mass index, waist circumference, or elevated random blood sugar.

	All	BMI for Age Z-Score (WHO)				Waist Circumference (WC)			Random Blood Sugar (RBS)		
	Subjects	Normal	Overweight	Obese		Normal	Central Obesity		<140 mg/dL	≥140 mg/dL	
	N (% Col)	N (% Row)	N (% Row)	N (% Row)	*p*-Value	N (% Row)	N (% Row)	*p*-Value	N (% Row)	N (% Row)	*p*-Value
Did the adolescents ’s mother develop T2DM during pregnancy?											
No	363 (79.1)	211 (58.1)	85 (23.4)	67 (18.5)	0.27	223 (61.9)	137 (38.1)	0.51	347 (95.6)	16 (4.4)	0.38
Yes	75 (16.3)	39 (52.0)	16 (21.3)	20 (26.7)		49 (66.2)	25 (33.8)		70 (93.3)	5 (6.7)	
Adolescents’ birth weight?											
<2.5 kg	44 (9.6)	30 (68.2)	4 (9.1)	10 (22.7)	0.68	30 (69.8)	13 (30.2)		40 (90.9)	4 (9.1)	0.07
2.5–4.0 kg	288 (62.8)	166 (57.6)	69 (24.0)	53 (18.4)		177 (62.1)	108 (37.9)	0.74	274 (95.1)	14 (4.9)	
≥4.0 kg	31 (6.8)	16 (51.6)	11 (35.5)	4 (12.9)		21 (67.7)	10 (32.3)		31 (100.0)	0 (0.0)	
Was the adolescent breast-fed?											
No	25 (5.5)	11 (44.0)	3 (12.0)	11 (44.0)	**0.0007**	14 (56.0)	11 (44.0)	0.49	24 (96.0)	1 (4.0)	0.57
<6 months	86 (18.7)	43 (50.0)	17 (19.8)	26 (30.2)		53 (61.6)	33 (38.4)		83 (96.5)	3 (3.5)	
≥6 months	304 (66.2)	183 (60.2)	75 (24.7)	46 (15.1)		192 (64.0)	108 (36.0)		289 (95.1)	15 (4.9)	
Do the adolescent’s siblings have T2DM?											
No	401 (87.4)	233 (58.1)	90 (22.4)	78 (19.5)	0.07	250 (63.0)	147 (37.0)	0.06	382 (95.3)	19 (4.7)	0.06
Yes	8 (1.7)	3 (37.5)	1 (12.5)	4 (50.0)		8 (100.0)	0 (0.0)		6 (75.0)	2 (25.0)	
Does the adolescent’s mother have T2DM?											
No	385 (83.9)	223 (57.9)	93 (24.2)	69 (17.9)	**0.02**	239 (62.7)	142 (37.3)	1.00	366 (95.1)	19 (4.9)	0.15
Yes	39 (8.5)	19 (48.7)	4 (10.3)	16 (41.0)		25 (64.1)	14 (35.9)		35 (83.7)	4 (10.3)	
Does the adolescent’s father have T2DM?											
No	322 (70.2)	194 (60.3)	74 (23.0)	54 (16.8)	**0.003**	201 (63.2)	117 (36.8)	0.73	305 (94.7)	17 (5.3)	1.00
Yes	111 (24.2)	51 (46.0)	28 (25.2)	32 (28.8)		68 (61.3)	43 (38.7)		105 (94.6)	6 (5.4)	
Any first-degree relatives with T2DM?											
No	295 (64.3)	179 (60.7)	70 (23.7)	46 (15.6)	**0.001**	184 (63.2)	107 (36.8)	0.91	280 (94.9)	15 (5.1)	1.00
Yes	135 (29.4)	65 (48.2)	30 (22.2)	40 (29.6)		84 (62.2)	51 (37.8)		128 (94.8)	7 (5.2)	
Do any of the mother’s relatives have T2DM?											
No	155 (33.8)	98 (63.2)	35 (22.6)	22 (14.2)	**0.01**	102 (66.2)	52 (33.8)	0.42	147 (94.8)	8 (5.2)	0.82
Yes	274 (59.7)	144 (52.6)	66 (24.1)	64 (23.4)		169 (62.4)	102 (37.6)		261 (95.3)	13 (4.7)	
Do any of the father’s relatives have T2DM?											
No	139 (30.3)	79 (56.8)	34 (24.5)	26 (18.7)	0.84	86 (62.3)	52 (37.7)	0.70	128 (92.1)	11 (7.9)	0.11
Yes	291 (63.4)	165 (56.7)	67 (23.0)	59 (20.3)		185 (64.2)	103 (35.8)		279 (95.9)	12 (4.1)	
Mother’s BMI											
Normal weight	107 (23.3)	71 (66.4)	21 (19.6)	15 (14.0)	**0.004**	76 (73.1)	28 (26.9)	**0.007**	103 (96.3)	4 (3.7)	0.57
Overweight	146 (31.8)	88 (60.3)	37 (25.3)	21 (14.4)		92 (63.5)	53 (36.6)		136 (93.2)	10 (6.8)	
Obese	97 (21.1)	46 (47.4)	23 (23.7)	28 (28.9)		53 (54.6)	44 (45.4)		92 (94.9)	5 (5.2)	
Father’s BMI											
Normal weight	74 (16.1)	48 (64.9)	12 (16.2)	14 (18.9)	0.08	50 (69.4)	22 (30.6)	0.19	71 (96.0)	3 (4.0)	0.35
Overweight	167 (36.4)	102 (61.1)	38 (22.8)	27 (16.2)		104 (62.7)	62 (37.3)		159 (95.2)	8 (4.8)	
Obese	97 (21.1)	49 (50.5)	24 (24.7)	24 (24.7)		57 (59.4)	39 (40.6)		90 (92.8)	7 (7.2)	
Is acanthosis nigricans present?											
No	389 (84.7)	239 (61.6)	95 (24.5)	54 (13.9)	**<0.0001**	252 (65.3)	134 (34.7)	**0.0005**	373 (95.9)	16 (4.1)	0.04
Yes	56 (12.2)	11 (16.6)	10 (17.9)	35 (62.5)		23 (41.1)	33 (58.9)		50 (89.3)	6 (10.7)	

Percentages may not add to 100% as data are missing for some variables (number of missing values: “Did the adolescent’s mother develop T2DM during pregnancy?” *n* = 21; “Adolescent’s birth weight?” *n* = 96; “Was the adolescent breastfed?” *n* = 44; “Do the adolescent’s siblings have T2DM?” *n* = 50; ”Does the adolescent’s mother have T2DM?” *n* = 35; “Does the adolescent’s father have T2DM?” *n* = 26; “Any first degree relatives with T2DM?” *n* = 29; ”Do any of the mother’s relatives have T2DM?” *n* = 30; ”Do any of the father’s relatives have T2DM?” *n* = 29; “Mother’s BMI” *n* = 109; “Father’s BMI” *n* = 121; “Is acanthosis nigricans present?” *n* = 14). *p*-values were calculated using the Chi-square test or the Fisher’s exact test for categorical variables (WC and RBS) and the Mantel–Haenszel test for trend for ordinal variables (BMI). Significant *p*-values are reported in bold format.

**Table 3:** Predictor of adolescent overweight, obesity, central obesity, and prediabetes at multivariable analysis.

	BMI for Age Z-Score (WHO)				Waist Circumference (WC)		Random Blood Sugar (RBS)	
	Overweight		Obesity		Central Obesity		≥140 mg/dL	
	OR (95% CI)	*p*	OR (95% CI)	*p*	OR (95% CI)	*p*	OR (95% CI)	*p*
**Gender**								
Boys	1.00		1.00		1.00		1.00	
Girls	0.76 (0.47–1.21)	0.25	0.71 (0.39–1.28)	0.25	**3.87 (2.47–6.07)**	<0.0001	1.44 (0.57–3.63)	0.44
**Country of citizenship**								
Qatar	1.00		1.00		1.00		1.00	
Other Arab country	1.15 (0.61–2.19)	0.67	0.62 (0.29–1.32)	0.22	**2.13 (1.17–3.88)**	**0.01**	1.37 (0.42–4.46)	0.61
South Asia	1.19 (0.62–2.29)	0.61	**0.28 (0.12–0.70)**	**0.006**	**4.35 (2.35–8.04)**	**<0.0001**	0.83 (0.23–2.97)	0.77
East Asia and Pacific	0.62 (0.22–1.74)	0.36	2.33 (0.99–5.47)	0.053	**2.92 (1.34–6.38)**	**0.007**	2.48 (0.60–10.3)	0.21
Other countries	1.15 (0.31–4.26)	0.82	0.44 (0.08–2.50)	0.36	1.77 (0.47–6.72)	0.40	-	0.97
**Was the adolescent breastfed?**								
≥6 months	1.00		1.00		1.00		1.00	
<6 months	1.10 (0.57–2.11)	0.77	2.02 (0.99–4.10)	0.053	1.14 (0.65–1.99)	0.66	0.62 (0.16–2.34)	0.48
No	0.69 (0.18–2.63)	0.59	**3.17 (1.09–9.26)**	**0.03**	2.27 (0.89–5.78)	0.09	0.82 (0.10–6.90)	0.85
**Any first-degree relatives with diabetes?**								
No	1.00		1.00		1.00		1.00	
Yes	1.04 (0.61–1.78)	0.89	**2.27 (1.22–4.24)**	**0.01**	0.98 (0.61–1.59)	0.95	0.94 (0.35–2.53)	0.90
**Mother’s BMI**								
Normal weight	1.00		1.00		1.00		1.00	
Overweight	1.35 (0.71–2.57)	0.37	1.20 (0.50–2.88)	0.68	1.66 (0.90–3.09)	0.11	2.26 (0.63–8.14)	0.21
Obese	1.59 (0.77–3.28)	0.21	**2.40 (1.01–5.70)**	**0.048**	**2.21 (1.14–4.27)**	**0.02**	1.41 (0.33–5.95)	0.64
**Is acanthosis nigricans present?**								
No	1.00		1.00		1.00		1.00	
Yes	2.37 (0.94–5.96)	0.06	**19.8 (8.38–46.9)**	**<0.0001**	**3.67 (1.88–7.18)**	**0.0001**	**4.06 (1.41–11.7)**	**0.009**

Odds ratios (OR) and 95% confidence intervals (CI) obtained from multivariable logistic regression model with all variables presented in the table fitted simultaneously. Significant results are reported in bold format.

## Data Availability

The data presented in this study are available on request from the corresponding author. The data are not publicly available due to reasons of privacy.

## Supplementary Materials

The following supporting information can be downloaded at: https://www.mdpi.com/article/10.3390/ijerph192114601/s1, Supplementary Checklist: STROBE Statement—Checklist of items that should be included in reports of cross-sectional studies; Figure S1: Distribution of random blood sugar according to adolescents’ characteristics; Table S1: Association between diet pattern and adolescents’ overweight, obesity, central obesity, and elevated random blood sugar; Table S2: Association between food consumption and adolescents’ overweight, obesity, central obesity, and elevated random blood sugar; Table S3: Association between physical activity and sedentary behavior and adolescents’ overweight, obesity, central obesity, and elevated random blood sugar.
