# Supplementary material for: Assessment of Overweight, Obesity, Central Obesity, and Type 2 Diabetes among Adolescents in Qatar: A Cross-Sectional Study
Source: Int J Environ Res Public Health. 2022 Nov 7;19(21):14601. doi: 10.3390/ijerph192114601 (PMC9653877; doi:10.3390/ijerph192114601)
Supplement: Supplementary file 1 [file ijerph-19-14601-s001.zip › ijerph-1978506-supplementary.pdf]

Supplementary checklist: STROBE Statement—Checklist of items that should be included in reports of ***cross-sectional studies***

	Item No	Recommendation	Pages
**Title and abstract**	1	(*a*) Indicate the study's design with a commonly used term in the title or the abstract	2
		(*b*) Provide in the abstract an informative and balanced summary of what was done and what was found	2
**Introduction**			
Background/rationale	2	Explain the scientific background and rationale for the investigation being reported	3-4
Objectives	3	State specific objectives, including any prespecified hypotheses	4
**Methods**			
Study design	4	Present key elements of study design early in the paper	4
Setting	5	Describe the setting, locations, and relevant dates, including periods of recruitment, exposure, follow-up, and data collection	4
Participants	6	(*a*) Give the eligibility criteria, and the sources and methods of selection of participants	4
Variables	7	Clearly define all outcomes, exposures, predictors, potential confounders, and effect modifiers. Give diagnostic criteria, if applicable	4-6
Data sources/ measurement	8*	For each variable of interest, give sources of data and details of methods of assessment (measurement). Describe comparability of assessment methods if there is more than one group	6
Bias	9	Describe any efforts to address potential sources of bias	4-5
Study size	10	Explain how the study size was arrived at	4
Quantitative variables	11	Explain how quantitative variables were handled in the analyses. If applicable, describe which groupings were chosen and why	6
Statistical methods	12	(*a*) Describe all statistical methods, including those used to control for confounding	6
		(*b*) Describe any methods used to examine subgroups and interactions	6
		(*c*) Explain how missing data were addressed	6
		(*d*) If applicable, describe analytical methods taking account of sampling strategy	6
		(*e*) Describe any sensitivity analyses	NA
**Results**			
Participants	13*	(a) Report numbers of individuals at each stage of study—eg numbers potentially eligible, examined for eligibility, confirmed eligible, included in the study, completing follow-up, and analysed	6-7
		(b) Give reasons for non-participation at each stage	NA
		(c) Consider use of a flow diagram	NA
Descriptive data	14*	(a) Give characteristics of study participants (eg demographic, clinical, social) and information on exposures and potential confounders	6-7
		(b) Indicate number of participants with missing data for each variable of interest	Tables 1-2
Outcome data	15*	Report numbers of outcome events or summary measures	Table 1

Main results	16	(*a*) Give unadjusted estimates and, if applicable, confounder-adjusted estimates and their precision (eg, 95% confidence interval). Make clear which confounders were adjusted for and why they were included	Table 1
		(*b*) Report category boundaries when continuous variables were categorized	4<br>Tables 2-3
		(*c*) If relevant, consider translating estimates of relative risk into absolute risk for a meaningful time period	NA
Other analyses	17	Report other analyses done—eg analyses of subgroups and interactions, and sensitivity analyses	Tables 2-3
**Discussion**			
Key results	18	Summarise key results with reference to study objectives	8
Limitations	19	Discuss limitations of the study, taking into account sources of potential bias or imprecision. Discuss both direction and magnitude of any potential bias	10
Interpretation	20	Give a cautious overall interpretation of results considering objectives, limitations, multiplicity of analyses, results from similar studies, and other relevant evidence	8-10
Generalisability	21	Discuss the generalisability (external validity) of the study results	10
**Other information**			
Funding	22	Give the source of funding and the role of the funders for the present study and, if applicable, for the original study on which the present article is based	10

*Give information separately for exposed and unexposed groups.

Note: An Explanation and Elaboration article discusses each checklist item and gives methodological background and published examples of transparent reporting. The STROBE checklist is best used in conjunction with this article (freely available on the Web sites of PLoS Medicine at http://www.plosmedicine.org/, Annals of Internal Medicine at http://www.annals.org/, and Epidemiology at http://www.epidem.com/). Information on the STROBE Initiative is available at www.strobe-statement.org.

Supplementary Figure S1. Distribution of random blood sugar according to adolescents' characteristics

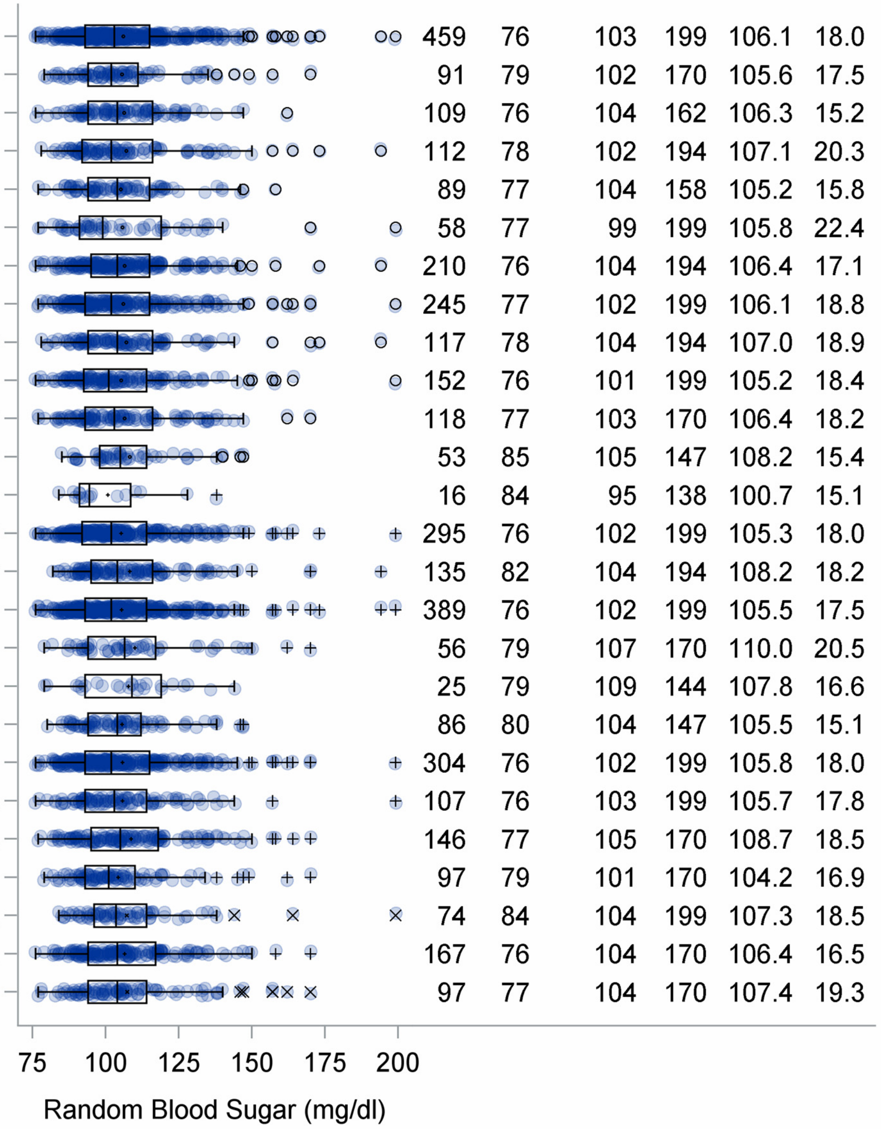

	N	Min	Median	Max	Mean	SD	P-value
All children	459	76	103	199	106.1	18.0	-
13 years old	91	79	102	170	105.6	17.5	Reference
14 years old	109	76	104	162	106.3	15.2	0.77
15 years old	112	78	102	194	107.1	20.3	0.57
16 years old	89	77	104	158	105.2	15.8	0.87
17 years old	58	77	99	199	105.8	22.4	0.95
Boys	210	76	104	194	106.4	17.1	Reference
Girls	245	77	102	199	106.1	18.8	0.83
Qatar	117	78	104	194	107.0	18.9	Reference
Other Arab countries	152	76	101	199	105.2	18.4	0.44
South Asian country	118	77	103	170	106.4	18.2	0.81
East Asian country	53	85	105	147	108.2	15.4	0.68
Other countries	16	84	95	138	100.7	15.1	0.20
No DM in family	295	76	102	199	105.3	18.0	Reference
DM in family	135	82	104	194	108.2	18.2	0.12
No acanthosis nigricans	389	76	102	199	105.5	17.5	Reference
Acanthosis nigricans	56	79	107	170	110.0	20.5	0.08
No Breast fed	25	79	109	144	107.8	16.6	Reference
Breast-fed <6 months	86	80	104	147	105.5	15.1	0.53
Breast-fed >6 months	304	76	102	199	105.8	18.0	0.60
Mother normal weight	107	76	103	199	105.7	17.8	Reference
Mother overweight	146	77	105	170	108.7	18.5	0.20
Mother obese	97	79	101	170	104.2	16.9	0.55
Father normal weight	74	84	104	199	107.3	18.5	Reference
Father overweight	167	76	104	170	106.4	16.5	0.72
Father obese	97	77	104	170	107.4	19.3	0.97

P-values obtained from the Student T-test

Supplementary Table S1. Association between diet pattern and adolescents' overweight, obesity, central obesity and elevated random blood sugar

	All	BMI for age Z-score (WHO)				Waist circumference (WC)			Random Blood Sugar (RBS)		
	Subjects	Normal	Overweight	Obese		Normal	Central obesity		<140 mg/dl	≥140 mg/dl	
	N (% col)	N (% row)	N (% row)	N (% row)	P-value	N (% row)	N (% row)	P-value	N (% row)	N (% row)	P-value
**All adolescents**	459 (100)	260 (56.8)	107 (23.4)	91 (19.9)		284 (62.4)	171 (37.6)		436 (95.0)	23 (5.0)	
How often do you Eat Breakfast											
Less than daily	229 (49.9)	126 (55.0)	52 (22.7)	51 (22.3)	0.27	133 (58.8)	93 (41.2)	0.11	221 (96.5)	8 (3.5)	0.14
Daily	228 (49.7)	133 (58.6)	54 (23.8)	40 (17.6)		150 (66.1)	77 (33.9)		213 (93.4)	15 (6.6)	
How often do you Eat Lunch											
Less than daily	102 (22.2)	59 (57.8)	15 (14.7)	28 (27.5)	0.33	62 (61.4)	39 (38.6)	0.81	99 (97.1)	3 (2.9)	0.44
Daily	354 (77.1)	201 (56.9)	89 (25.2)	63 (17.8)		220 (62.7)	131 (37.3)		334 (94.4)	20 (5.6)	
How often do you Eat Dinner											
Less than daily	199 (43.4)	108 (54.3)	47 (23.6)	44 (22.1)	0.28	115 (58.4)	82 (41.6)	0.09	191 (96.0)	8 (4.0)	0.51
Daily	251 (54.7)	147 (58.8)	57 (22.8)	46 (18.4)		165 (66.3)	84 (33.7)		237 (94.4)	14 (5.6)	
How often do you Snack between meals											
Never	41 (8.9)	22 (53.7)	8 (19.5)	11 (26.8)	0.14	19 (46.3)	22 (53.7)	0.12	40 (97.6)	1 (2.4)	**0.04**
1-3 times/week	197 (42.9)	109 (55.3)	46 (23.4)	42 (21.3)		122 (62.6)	73 (37.4)		191 (97.0)	6 (3.0)	
4-6 times/week	101 (22.0)	55 (55.0)	26 (26.0)	19 (19.0)		67 (66.3)	34 (33.7)		94 (93.1)	7 (6.9)	
Daily	118 (25.7)	73 (61.9)	26 (22.0)	19 (16.1)		75 (64.7)	41 (35.3)		109 (92.4)	9 (7.6)	
How often do you Eat while watching TV											
Never	100 (21.8)	61 (61.6)	23 (23.2)	15 (15.2)	0.06	58 (58.6)	41 (41.4)	0.76	97 (97.0)	3 (3.0)	0.28
1-3 times/week	170 (37.0)	99 (58.2)	40 (23.5)	31 (18.2)		111 (65.7)	58 (34.3)		161 (94.7)	9 (5.3)	
4-6 times/week	89 (19.4)	52 (58.4)	13 (14.6)	24 (27.0)		60 (68.2)	28 (31.8)		86 (96.6)	3 (3.4)	
Daily	97 (21.1)	47 (48.5)	29 (29.9)	21 (21.6)		54 (56.3)	42 (43.8)		90 (92.8)	7 (7.2)	
How often do you Eat when bored											
Never	155 (33.8)	92 (59.7)	32 (20.8)	30 (19.5)	0.23	101 (65.2)	54 (34.8)	0.73	147 (94.8)	8 (5.2)	0.86
1-3 times/week	152 (33.1)	72 (47.4)	45 (29.6)	35 (23.0)		84 (56.0)	66 (44.0)		144 (94.7)	8 (5.3)	

4-6 times/week	79 (17.2)	48 (60.8)	18 (22.8)	13 (16.5)		53 (67.1)	26 (32.9)		77 (97.5)	2 (2.5)	
Daily	69 (15.0)	47 (68.1)	11 (15.9)	11 (15.9)		44 (65.7)	23 (34.3)		64 (97.0)	2 (3.0)	
How often do you Eat when stressed											
Never	299 (65.1)	167 (56.0)	76 (25.5)	55 (18.5)		188 (62.9)	111 (37.1)		284 (95.0)	15 (5.0)	
1-3 times/week	97 (21.1)	61 (62.9)	18 (18.6)	18 (18.6)	0.45	60 (63.2)	35 (36.8)	0.57	93 (95.9)	4 (4.1)	0.44
4-6 times/week	37 (8.1)	16 (43.2)	10 (27.0)	11 (29.7)		23 (63.9)	13 (36.1)		36 (97.3)	1 (2.7)	
Daily	22 (4.8)	14 (63.6)	2 (9.1)	6 (27.3)		11 (52.4)	10 (47.6)		19 (86.4)	3 (13.6)	
How often do you Eat at late hours of night											
Never	132 (28.8)	80 (60.6)	34 (25.8)	18 (13.6)		79 (60.3)	52 (39.7)		126 (94.0)	8 (6.0)	
1-3 times/week	197 (42.9)	110 (56.1)	45 (23.0)	41 (20.9)	0.30	124 (63.3)	72 (36.7)	0.49	190 (96.4)	7 (3.6)	**0.04**
4-6 times/week	80 (17.4)	39 (48.8)	18 (22.5)	23 (28.8)		50 (63.3)	29 (36.7)		77 (96.3)	3 (3.8)	
Daily	48 (10.5)	31 (64.6)	9 (18.8)	8 (16.7)		31 (66.0)	16 (34.0)		41 (85.4)	7 (14.6)	
How often do you Eat when angry											
Never	363 (79.1)	200 (55.1)	88 (24.2)	75 (20.7)		223 (61.8)	138 (38.2)		344 (94.8)	19 (5.2)	
1-3 times/week	63 (13.7)	42 (66.7)	12 (19.0)	9 (14.3)	0.34	43 (70.5)	18 (29.5)	0.92	60 (95.2)	3 (4.8)	0.75
4-6 times/week	19 (4.1)	9 (50.0)	4 (22.2)	5 (27.8)		10 (52.6)	9 (47.4)		19 (100.)	0 (0.0)	
Daily	13 (2.8)	9 (69.2)	2 (15.4)	2 (15.4)		8 (61.5)	5 (38.5)		12 (92.3)	1 (7.7)	

Supplementary Table S2. Association between food consumption and adolescents' overweight, obesity, central obesity and elevated random blood sugar

	All	BMI for age Z-score (WHO)				Waist circumference			Random Blood Sugar		
	Subjects	Normal	Overweight	Obese		Normal	Central obesity		<140 mg/dl	≥140 mg/dl	
	N (% col)	N (% row)	N (% row)	N (% row)	P-value	N (% row)	N (% row)	P-value	N (% row)	N (% row)	P-value
**All adolescents**	459 (100)	260 (56.8)	107 (23.4)	91 (19.9)		284 (62.4)	171 (37.6)		436 (95.0)	23 (5.0)	
**Fruit, such as apples, oranges, bananas or grapes**											
<1 time/day	140 (30.5)	78 (55.7)	32 (22.9)	30 (21.4)	0.85	88 (62.9)	52 (37.1)	0.80	131 (93.6)	9 (6.4)	0.07
1 time/day	142 (30.9)	88 (62.4)	31 (22.0)	22 (15.6)		91 (65.0)	49 (35.0)		131 (92.3)	11 (7.7)	
2 times/day	79 (17.2)	39 (49.4)	21 (26.6)	19 (24.1)		42 (53.2)	37 (46.8)		76 (96.2)	3 (3.8)	
3+ times/day	56 (12.2)	31 (55.4)	16 (28.6)	9 (16.1)		37 (67.3)	18 (32.7)		56 (100.)	0 (0.0)	
**Vegetables, such as tomatoes, cucumbers, or carrots**											
<1 time/day	120 (26.1)	74 (62.2)	25 (21.0)	20 (16.8)	0.55	79 (66.4)	40 (33.6)	0.51	117 (97.5)	3 (2.5)	0.95
1 time/day	145 (31.6)	80 (55.2)	38 (26.2)	27 (18.6)		90 (62.5)	54 (37.5)		134 (92.4)	11 (7.6)	
2 times/day	92 (20.0)	50 (54.3)	21 (22.8)	21 (22.8)		52 (57.1)	39 (42.9)		85 (92.4)	7 (7.6)	
3+ times/day	69 (15.0)	42 (60.9)	14 (20.3)	13 (18.8)		44 (64.7)	24 (35.3)		68 (98.6)	1 (1.4)	
**Cooked vegetables such as marrow, okra, or broccoli**											
<1 time/day	221 (48.1)	123 (55.9)	59 (26.8)	38 (17.3)	0.84	137 (62.8)	81 (37.2)	0.57	212 (95.9)	9 (4.1)	0.44
1 time/day	108 (23.5)	60 (55.6)	21 (19.4)	27 (25.0)		67 (62.0)	41 (38.0)		99 (91.7)	9 (8.3)	
2 times/day	44 (9.6)	23 (52.3)	13 (29.5)	8 (18.2)		21 (47.7)	23 (52.3)		43 (97.7)	1 (2.3)	
3+ times/day	37 (8.1)	22 (59.5)	8 (21.6)	7 (18.9)		24 (66.7)	12 (33.3)		37 (100.)	0 (0.0)	
**Starchy foods such as bread, pasta, rice, potatoes (including fries), pizza or Fatayer (pastries)**											
<1 time/day	57 (12.4)	26 (46.4)	20 (35.7)	10 (17.9)	0.26	32 (56.1)	25 (43.9)	0.21	54 (94.7)	3 (5.3)	0.98
1 time/day	142 (30.9)	79 (55.6)	33 (23.2)	30 (21.1)		87 (62.6)	52 (37.4)		134 (94.4)	8 (5.6)	
2 times/day	122 (26.6)	68 (55.7)	27 (22.1)	27 (22.1)		72 (59.5)	49 (40.5)		117 (95.9)	5 (4.1)	
3+ times/day	123 (26.8)	77 (62.6)	22 (17.9)	24 (19.5)		83 (67.5)	40 (32.5)		116 (94.3)	7 (5.7)	
**Carbonated soft drinks, such as Pepsi and Coca Cola (Exclude diet sodas)**											
<1 time/day	287 (62.5)	160 (55.9)	70 (24.5)	56 (19.6)	0.27	168 (58.9)	117 (41.1)	0.15	277 (96.5)	10 (3.5)	0.12
1 time/day	70 (15.3)	37 (52.9)	19 (27.1)	14 (20.0)		50 (72.5)	19 (27.5)		63 (90.0)	7 (10.0)	
2 times/day	33 (7.2)	24 (72.7)	6 (18.2)	3 (9.1)		22 (68.8)	10 (31.3)		31 (93.9)	2 (6.1)	
3+ times/day	40 (8.7)	24 (60.0)	9 (22.5)	7 (17.5)		26 (65.0)	14 (35.0)		37 (92.5)	3 (7.5)	
**Fresh fruit juices**											
<1 time/day	162 (35.3)	94 (58.0)	41 (25.3)	27 (16.7)	0.31	104 (65.0)	56 (35.0)	0.99	158 (97.5)	4 (2.5)	0.63
1 time/day	151 (32.9)	87 (58.0)	33 (22.0)	30 (20.0)		84 (56.0)	66 (44.0)		138 (91.4)	13 (8.6)	
2 times/day	56 (12.2)	30 (53.6)	11 (19.6)	15 (26.8)		42 (76.4)	13 (23.6)		52 (92.9)	4 (7.1)	
3+ times/day	51 (11.1)	27 (52.9)	14 (27.5)	10 (19.6)		30 (58.8)	21 (41.2)		50 (98.0)	1 (2.0)	

**Energy drinks, such as Red Bull or Monster**											
<1 time/day	385 (83.9)	218 (56.8)	92 (24.0)	74 (19.3)	0.94	235 (61.7)	146 (38.3)	0.25	364 (94.5)	21 (5.5)	0.72
1 time/day	23 (5.0)	11 (47.8)	4 (17.4)	8 (34.8)		16 (69.6)	7 (30.4)		23 (100.)	0 (0.0)	
2 times/day	15 (3.3)	9 (60.0)	4 (26.7)	2 (13.3)		12 (80.0)	3 (20.0)		15 (100.)	0 (0.0)	
3+ times/day	8 (1.7)	5 (62.5)	2 (25.0)	1 (12.5)		5 (62.5)	3 (37.5)		7 (87.5)	1 (12.5)	
**Milk or dairy products, such as cheese, yoghurt or butter**											
<1 time/day	87 (19.0)	48 (55.2)	21 (24.1)	18 (20.7)	0.96	51 (59.3)	35 (40.7)	0.89	85 (97.7)	2 (2.3)	0.90
1 time/day	162 (35.3)	95 (59.0)	34 (21.1)	32 (19.9)		106 (65.4)	56 (34.6)		151 (93.2)	11 (6.8)	
2 times/day	99 (21.6)	55 (55.6)	25 (25.3)	19 (19.2)		62 (63.9)	35 (36.1)		93 (93.9)	6 (6.1)	
3+ times/day	82 (17.9)	45 (54.9)	21 (25.6)	16 (19.5)		48 (59.3)	33 (40.7)		80 (97.6)	2 (2.4)	
**Snacks like chips, chocolates, biscuits, and candies**											
<1 time/day	120 (26.1)	61 (51.3)	25 (21.0)	33 (27.7)	**0.004**	68 (57.6)	50 (42.4)	0.07	117 (97.5)	3 (2.5)	0.22
1 time/day	123 (26.8)	66 (53.7)	33 (26.8)	24 (19.5)		81 (66.4)	41 (33.6)		117 (95.1)	6 (4.9)	
2 times/day	116 (25.3)	67 (57.8)	29 (25.0)	20 (17.2)		65 (56.0)	51 (44.0)		106 (91.4)	10 (8.6)	
3+ times/day	87 (19.0)	59 (67.8)	17 (19.5)	11 (12.6)		65 (75.6)	21 (24.4)		83 (95.4)	4 (4.6)	
**Food from a fast-food restaurant, such as McDonalds, Burger King, or KFC**											
<1 time/day	272 (59.3)	156 (57.4)	64 (23.5)	52 (19.1)	0.86	165 (60.7)	107 (39.3)	0.16	260 (95.6)	12 (4.4)	0.89
1 time/day	86 (18.7)	53 (61.6)	17 (19.8)	16 (18.6)		61 (72.6)	23 (27.4)		80 (93.0)	6 (7.0)	
2 times/day	27 (5.9)	16 (59.3)	5 (18.5)	6 (22.2)		19 (73.1)	7 (26.9)		26 (96.3)	1 (3.7)	
3+ times/day	25 (5.5)	14 (56.0)	5 (20.0)	6 (24.0)		16 (64.0)	9 (36.0)		24 (96.0)	1 (4.0)	
**Eggs or meat products (meat, lamb, chicken)**											
<1 time/day	67 (14.6)	36 (54.5)	16 (24.2)	14 (21.2)	0.29	44 (66.7)	22 (33.3)	0.98	65 (97.0)	2 (3.0)	0.96
1 time/day	188 (41.0)	102 (54.3)	47 (25.0)	39 (20.7)		113 (60.4)	74 (39.6)		176 (93.6)	12 (6.4)	
2 times/day	109 (23.7)	66 (60.6)	23 (21.1)	20 (18.3)		66 (61.1)	42 (38.9)		104 (95.4)	5 (4.6)	
3+ times/day	84 (18.3)	51 (60.7)	18 (21.4)	15 (17.9)		54 (65.1)	29 (34.9)		80 (95.2)	4 (4.8)	
**Fish**											
<1 time/day	248 (54.0)	145 (58.7)	59 (23.9)	43 (17.4)	0.36	155 (62.8)	92 (37.2)	0.89	238 (96.0)	10 (4.0)	0.99
1 time/day	109 (23.7)	57 (52.3)	26 (23.9)	26 (23.9)		63 (58.9)	44 (41.1)		101 (92.7)	8 (7.3)	
2 times/day	31 (6.8)	19 (61.3)	5 (16.1)	7 (22.6)		21 (67.7)	10 (32.3)		30 (96.8)	1 (3.2)	
3+ times/day	13 (2.8)	7 (53.8)	3 (23.1)	3 (23.1)		7 (58.3)	5 (41.7)		13 (100.)	0 (0.0)	

Supplementary Table S3. Association between physical activity and sedentary behavior and adolescents' overweight, obesity, central obesity and elevated random blood sugar

	**All**	**BMI for age Z-score (WHO)**				**Waist circumference (WC)**			**Random Blood Sugar (RBS)**		
	**Subjects**	**Normal**	**Overweight**	**Obese**		**Normal**	**Central obesity**		**<140 mg/dl**	**≥140 mg/dl**	
	**N (% col)**	**N (% row)**	**N (% row)**	**N (% row)**	**P-value**	**N (% row)**	**N (% row)**	**P-value**	**N (% row)**	**N (% row)**	**P-value**
**All adolescents**	459 (100)	260 (56.8)	107 (23.4)	91 (19.9)		284 (62.4)	171 (37.6)		436 (95.0)	23 (5.0)	
**In the last 7 days, how many days did you engage in any sport or fitness activity?**											
I did not engage in any PA	84 (18.3)	47 (56.6)	15 (18.1)	21 (25.3)		45 (54.9)	37 (45.1)		79 (94.0)	5 (6.0)	
1-2 days/week	212 (46.2)	122 (57.5)	47 (22.2)	43 (20.3)	0.30	126 (60.0)	84 (40.0)	**0.003**	199 (93.9)	13 (6.1)	0.20
3-4 days/week	86 (18.7)	45 (52.3)	23 (26.7)	18 (20.9)		53 (61.6)	33 (38.4)		83 (96.5)	3 (3.5)	
5-7 days/week	77 (16.8)	46 (59.7)	22 (28.6)	9 (11.7)		60 (77.9)	17 (22.1)		75 (97.4)	2 (2.6)	
**How much time per day do you engage in any sport or fitness activity?**											
I do not engage in any PA daily	90 (19.6)	54 (60.7)	15 (16.9)	20 (22.5)		57 (64.0)	32 (36.0)		87 (96.7)	3 (3.3)	
Less than 1/2 hour	104 (22.7)	60 (57.7)	21 (20.2)	23 (22.1)	0.93	56 (54.9)	46 (45.1)	0.27	93 (89.4)	11 (10.6)	0.28
1/2 hour-1 hour	155 (33.8)	90 (58.1)	33 (21.3)	32 (20.6)		95 (61.7)	59 (38.3)		149 (96.1)	6 (3.9)	
More than 1 hour	110 (24.0)	56 (50.9)	38 (34.5)	16 (14.5)		76 (69.1)	34 (30.9)		107 (97.3)	3 (2.7)	
**How many hours per day do you spend watching TV?**											
I do not watch TV	148 (32.2)	83 (56.5)	41 (27.9)	23 (15.6)		94 (64.8)	51 (35.2)		146 (98.6)	2 (1.4)	
0-1 hours/day	184 (40.1)	105 (57.1)	40 (21.7)	39 (21.2)	0.57	115 (62.5)	69 (37.5)	0.54	173 (94.0)	11 (6.0)	
2-4 hours/day	109 (23.7)	61 (56.0)	22 (20.2)	26 (23.9)		62 (57.4)	46 (42.6)		100 (91.7)	9 (8.3)	
5+ hours/day	18 (3.9)	11 (61.1)	4 (22.2)	3 (16.7)		13 (72.2)	5 (27.8)		17 (94.4)	1 (5.6)	**0.02**
**How many hours per day do you spend using a computer/tablet/iPad?**											
I do not use a computer/tablet/iPad	102 (22.2)	57 (55.9)	28 (27.5)	17 (16.7)		66 (66.0)	34 (34.0)		97 (95.1)	5 (4.9)	
0-1 hours/day	143 (31.2)	90 (63.4)	30 (21.1)	22 (15.5)		89 (63.1)	52 (36.9)	0.35	139 (97.2)	4 (2.8)	0.34
2-4 hours/day	136 (29.6)	71 (52.2)	35 (25.7)	30 (22.1)	0.10	83 (61.0)	53 (39.0)		127 (93.4)	9 (6.6)	
5+ hours/day	77 (16.8)	42 (54.5)	13 (16.9)	22 (28.6)		46 (59.7)	31 (40.3)		72 (92.3)	6 (7.7)	
**How many hours per day do you spend using your mobile phone?**											
I do not use a mobile phone	42 (9.2)	22 (52.4)	12 (28.6)	8 (19.0)		20 (47.6)	22 (52.4)		42 (100.)	0 (0.0)	
0-1 hours/day	94 (20.5)	60 (64.5)	24 (25.8)	9 (9.7)	0.11	52 (56.5)	40 (43.5)	**0.03**	86 (91.5)	8 (8.5)	0.47
2-4 hours/day	160 (34.9)	89 (55.6)	38 (23.8)	33 (20.6)		106 (67.1)	52 (32.9)		149 (93.1)	11 (6.9)	
5+ hours/day	163 (35.5)	89 (54.6)	33 (20.2)	41 (25.2)		106 (65.0)	57 (35.0)		159 (97.5)	4 (2.5)	

**How many hours per day do you spend watching TV, using a computer/tablet/iPad or a mobile phone.**											
0-3 hours/day	95 (20.7)	61 (64.9)	25 (26.6)	8 (8.5)	**0.005**	55 (59.1)	38 (40.9)	0.26	92 (96.8)	3 (3.2)	0.53
4-5 hours/day	102 (22.2)	57 (55.9)	29 (28.4)	16 (15.7)		57 (56.4)	44 (43.6)		98 (96.1)	4 (3.9)	
6-7 hours/day	116 (25.3)	63 (54.3)	25 (21.6)	28 (24.1)		80 (69.6)	35 (30.4)		106 (91.4)	10 (8.6)	
8+ hours/day	146 (31.8)	79 (54.1)	28 (19.2)	39 (26.7)		92 (63.0)	54 (37.0)		140 (95.9)	6 (4.1)	
